# A randomized, placebo-controlled clinical trial evaluating of a mouthwash containing *Sambucus williamsii* var. *coreana* extract for prevention of gingivitits

**DOI:** 10.1038/s41598-022-15445-7

**Published:** 2022-07-18

**Authors:** Yu-Rin Kim, Seoul-Hee Nam

**Affiliations:** 1 Department of Dental Hygiene, Silla University, 140 Baegyang-daero 700beon-gil, Sasang-gu, Busan, 46958 Republic of Korea; 2 Department of Dental Hygiene, College of Health Sciences, Kangwon National University, 346 Hwangjo-gil, Dogye-up, Samcheok-si, Gangwon-do 25945 Republic of Korea

**Keywords:** Plant sciences, Health care

## Abstract

The purpose of this study is to verify the clinical applicability by applying a mouthwash containing *Sambucus williamsii* var. *coreana* extract for preventing periodontal disease. A randomized, double-blind, placebo-controlled study was conducted on 64 patients, excluding those with insufficient data, who visited M dental clinic located in Busan, Korea. Thirty-two people were assigned respectively to the saline solution gargle group and the *Sambucus williamsii* var. *coreana* extract gargle group to conduct the O'Leary index, plaque index (PI), gingival index (GI), and subgingival plaques. For the homogeneity of the two groups, scaling was carried out one week before the experiment, and the participants were taught for oral care to conduct during the study period. SPSS 24.0 for Windows (IBM Corp., Armonk, NY, USA) was used to compare the saline solution gargle group and the *Sambucus williamsii* var. *coreana* extract gargle group as well as to analyze Baseline (before gargle application), Treatment (immediately after gargle application), and After 5 Days (5 days after gargle application). There was a significant difference in the O'Leary index, PI, GI and subgingival plaques after Treatment and After 5 days (*p* < 0.05). Also, the periodontal-related indexes improved as the application time increased in the *Sambucus williamsii* var. *coreana* extract gargle group. The antibacterial effect was also shown for gram-positive bacteria and gram-negative bacteria in subgingival plaques as the application time increased. The use of the mouthwash containing *Sambucus williamsii* var*. coreana* extract was found to be effective for oral periodontal-related indicators and bacteria causing periodontal disease. Therefore, using a mouthwash containing *Sambucus williamsii* var. *coreana* extract, a natural drug, will possibly maintain healthy periodontal health by inhibiting and preventing the progression of periodontal disease.

## Introduction

Periodontal disease is a non-communicable chronic disease that destroys soft and hard tissues due to local bacterial infection and imbalanced host immune responses^1^. As periodontal disease progresses, the collagen fibers of the periodontal ligament are destroyed to form a periodontal pocket, which creates a favorable ecological environment for anaerobic bacteria to thrive^2^. Most periodontal disease bacteria are anaerobic and sensitive to oxygen, so they stay in the subgingival biofilm, where oxygen supply is poor than in the supragingival biofilm^3^. The anaerobic bacteria associated with periodontal disease include *Parvimonas micra (P. micra)*, *Porphyromonas gingivalis (P. gingivalis)*, *Aggregatibacter actinomycetemcomitans (A. actinomycetemcomitans)*, *Treponema denticola (T. denticola)*, *Prevotella nigrescens (P. nigrescens)*, *Fusobacterium nucleatum (F. nucleatum)*^4^.

The lipopolysaccharides (LPS) endotoxins or metabolites formed by these anaerobic bacteria increase the secretion of proinflammatory cytokines from tissues and immune cells^5^. In particular, Interleukin-1 (IL-1β) and Tumor necrosis factor-α (TNF-α) are representative cytokines involved in tissue destruction. IL-1β induces the followings: recruitment of inflammatory cells, priming degranulation of polymorphonuclear leukocytes, production of inflammatory mediators such as prostaglandin, production of matrix metalloproteinases (MMP), inhibiting collagen synthesis, activating T and B lymphocytes^6^. TNF-α increases cellular apoptosis, bone resorption, MMP secretion, intercellular adhesion molecule (ICAM) expression, and IL-6 production^7^. In addition, IL-6 is involved in the tissue destruction process by promoting osteoclast formation, bone resorption, and T lymphocyte differentiation^8^. Therefore, it is crucial to suppress anaerobic bacteria as soon as possible to prevent periodontal disease.

When inhibiting bacteria, mouthwash, one of the chemical plaque control practices, is becoming popular as it is easier to carry and use than toothbrushing, a mechanical and cumbersome way of controlling plaque^9^. Above all, the SARS-CoV-2 virus of COVID-19, a cause of the pandemic, was found in 91.7% of saliva samples of patients with COVID-19^10^. Accordingly, mouthwash is recommended as an effective measure to reduce the risk of viral infection, and people's attention to mouthwash increases^11^—however, most mouthwashes with intensive chemical agents remove resident bacteria, not only pathogens in the mouth. Therefore, natural extracts are recommended rather than synthetic chemical agents to suppress only pathogens while maintaining healthy oral bacteria^12^.

Nowadays, many studies on natural extracts have been actively ongoing; practical use of natural antibacterial action varies accordingly^13–16^. Studies on *Glycyrrhiza uralensis* extract^17^ and *bamboo* extract^18^ said that these are effective natural agents showing antibacterial effects related to periodontal disease. *Onion* extract also inhibits strains of periodontal pathogens *P. gingivalis* and *Prevotella intermedia (P. intermedia)* at a concentration of 40 μg/ml^19^. In addition, *pulsatilla* extract has an antibacterial effect in the layer separated by butyl alcohol^20^. Asafoetida mouthwash has been studied as an effective mouthwash to improve the gingival health index^15^, and saffron mouthwash has been reported to have an anti-inflammatory effect in the treatment of periodontitis patients and to improve the periodontal period^16^. Even though researches on the antibacterial effects of various natural medicines continue, clinical studies about natural medicines preventing periodontal disease are insufficient. Therefore, it is necessary to prove the clinical effects of natural medicines to develop them as mouthwashes.

Williams Elder (*Sambucus williamsii* var. *coreana*) is a deciduous broad-leaved tree distributed in Korea, China, and Japan. This tree has been helpful as medicine for bones and musculoskeletal injuries in traditional oriental medicine^21^. There was a study of triterpenes, phenols, and lignans isolated from *Sambucus williamsii* var. *coreana* used in proliferation experiments on mouse osteocytes (UMR106) and alkaline phosphatase (ALP) activity experiments on UMR106 cells^22^. Based on the research results related to osteogenesis, *Sambucus williamsii* var. *coreana* possibly regenerates destroyed alveolar bones and periodontal tissues caused by periodontal disease. In terms of a study checking the relationship between *Sambucus williamsii* var. *coreana* and oral disease, there was a study showing the antibacterial effect of *Sambucus williamsii* var*. coreana* extract against oral pathogens^23^. However, no studies in the dental field verified the clinical usefulness of *Sambucus williamsii* var. *coreana* based on clinical effectiveness.

Therefore, we evaluated the clinical applicability of mouthwash using *Sambucus williamsii* var. *coreana* extract, a phytotherapeutic medication, to develop an effective medicine against periodontal-related clinical indicators and pathogens that cause periodontal disease.

## Materials and methods

### Ethical consideration

This study followed the International Council for Harmonization of Technical Requirements for Pharmaceuticals for human use (ICH) guideline. The Silla University Institutional Review Board (1041449-202008-HR-001, Busan, Korea) had approved the human study and WHO International Clinical Trial Registry Platform was registered by clinical trial registration (10/03/2022, registration number: KCT0007064; https://cris.nih.go.kr/cris/search/detailSearch.do/21436). All participants had noticed relevant information (purpose, procedures, and risks) of this study. Participants were free to withdraw from the study at any time. An informed consent form was provided to all participants prior to enrollment in the clinical trial and written informed consent was obtained.

### Study participants

**Figure 1:**
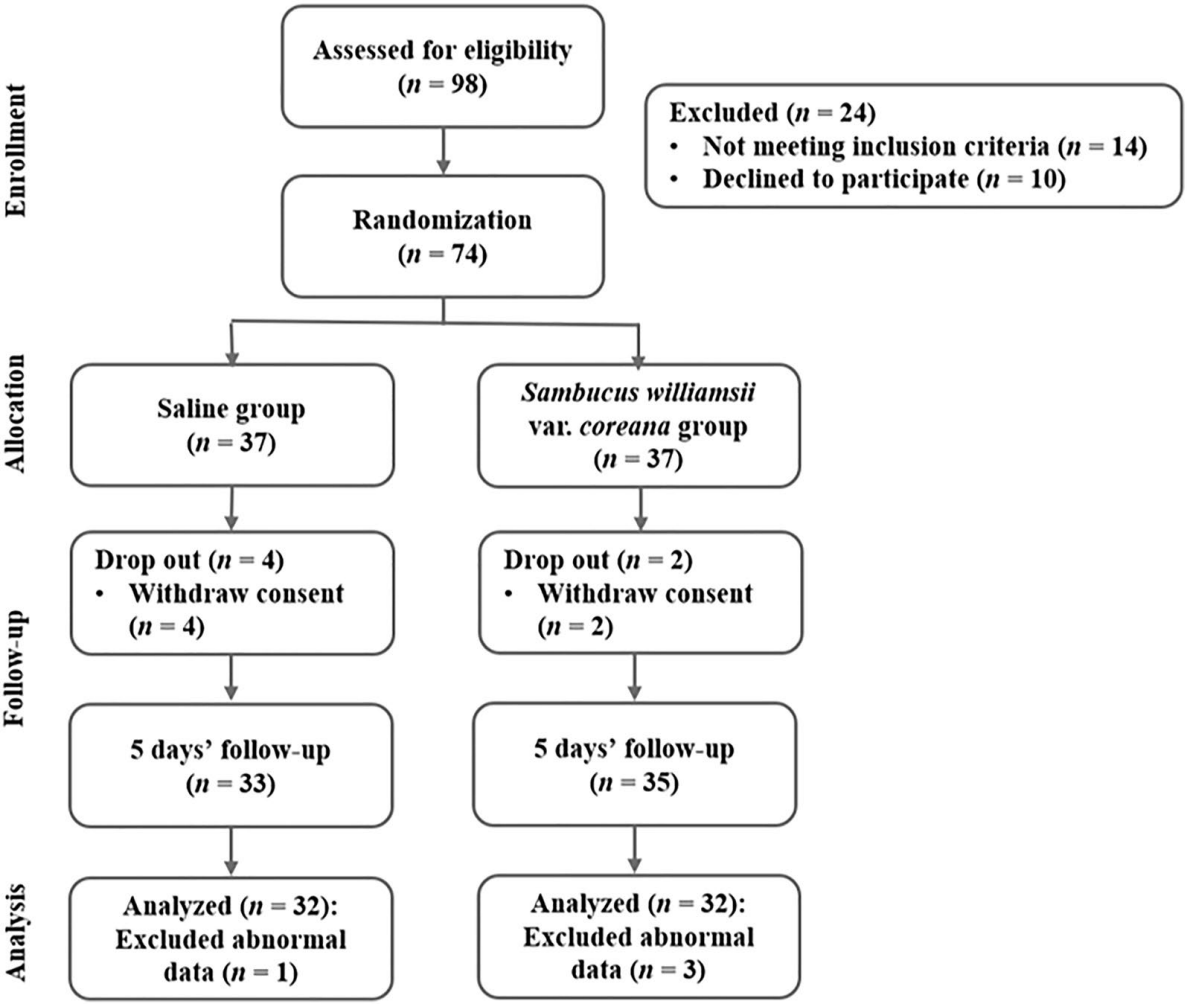
Flowchart of the study.

The G* Power 3.1 program calculated sample sizes. 68 participants were required for an independent t-test with significance level a = 0.05 two-tailed, power = 0.8, and effect size = 0.7. An initially planned sample size was 96 to consider a dropout rate of 40%, and actual participants were 98 in this study. The dropout rate was set high as the subjects were college students or working adults. 98 subjects were screened, but 74 participants were randomly assigned to the saline group or *Sambucus williamsii* var*. coreana* extract group after excluding 24 subjects who did not meet inclusion criteria or refused to participate during 5 days. 64 subjects were selected as final subjects, excluding 6 subjects who did not follow the guidelines for 5 days and 4 subjects who did not complete the final analysis (Fig. 1).

### Study design and protocol

An experiment was randomized, double-blind, and placebo-controlled. In this study, a dental hygienist with more than ten years of experience directly stated the aim of the study to the patients who visited M dental clinic in Busan from October 2020 to June 2021. These patients agreed to participate in the study on December 15, 2020. Among the subjects, the following subjects who met the exclusion criteria were excluded from this study: a person with severe dental disease (e.g., periodontitis, dry mouth, dental caries), a person with a disease that may cause bad breath (e.g., Sjogren's syndrome, rheumatism, renal disease, and hepatic disease), a smoking person, a person diagnosed with sinusitis or rhinitis, a person taking antibiotics, a person with tongue problems (e.g., tongue cancer, glossitis), a person received scaling within 2 months. Among dental caries, patients with enamel caries were eligible to participate in the study, but patients with more than one dentin caries were excluded. Accordingly, the subjects of this study were those who agreed to complete the questionnaire, were not included in the exclusion criteria, and had 16 or more remaining teeth. 74 patients who satisfied the inclusion criteria were selected as the study subjects. Finally, the participants in this study were analyzed with a result of 64 subjects.

### Clinical examination

Participants who agreed to this study visited the M dental clinic in Busan one week before the start and received an oral examination by a dentist. Two dental hygienists trained under the guidance of a dentist performed light scaling on the participants for the same oral environment and provided the same toothbrush and toothpaste to them. After a recovery period of 1 week after scaling, the study started. As representative teeth for an oral examination, maxillary right first molar (#16), maxillary left central incisor (#21), maxillary left first premolar (#24), mandibular left first molar (# 36), mandibular right central incisor (#41), and mandibular right first premolar (#44) were selected and measured.

After scaling, two dental hygienists trained under the guidance of a dentist obtained the following data: before the application of mouthwash as Baseline, immediately after the application of mouthwash as Treatment, and five days after the application of mouthwash as After 5 Days. Participants were provided with labeled items so that they could not tell whether they were in the experimental group or the control group. They were also educated on how to use mouthwash and how often and how to brush their teeth using the provided toothpaste and toothbrush. For five days, the experimental group held 15 ml of mouthwash containing *Sambucus williamsii* var. *coreana* extract for 30 s and then spat it out before going to bed after brushing, while the control group did the same with 15 ml of saline. After gargling, intake of food, including water, was prohibited. O'Leary index, plaque index (PI), gingival index (GI), and microbiological analysis were evaluated as indicators related to periodontal disease.

#### O’Leary index

We conducted O'Leary, Drake, and Naylor’s dental plaque test (O'Leary index)^24^, where all teeth in the oral cavity were discolored with a dental surface discoloration agent and estimated the status of adherence (%) using the plaque control score (O'Leary index). Score 1 if the dental plaque adheres to the dental surfaces of 4 teeth (mesial, efferent, facies, and lingual), and score 0 if not.

#### PI

Using the PI technique of Loe and Silness^25^, dividing all teeth surface into two parts; the gingival margin and the tooth surface. Then, measuring plaque accumulation and its thickness with a red colorant. The evaluation criteria are as follows; 0 = no plaque; 1 = thin plaque attached to the gingival margin and visible when rubbing gently with a probe or applying a tooth colorant; 2 = moderate plaque visible along the gingival margin; 3 = thick plaque collection in gingival margin and tooth surface as well as in the gingival pockets. PI is the average quantity of plaque per measured tooth surface, and its score for each tooth was calculated using the average value.

#### GI

Gingival health status at the proximal, distal, buccal, and lingual regions of all teeth was evaluated using the GI technique^26^. Each section was assigned from score 0 to 3: 0 = normal gingiva; 1 = a slight color change and swelling of gingivitis showing no bleeding with a mild stimulus; 2 = red and swollen gingivitis showing bleeding with a mild stimulus; 3 = remarkably red and swollen gingivitis due to progressed inflammation and showing the possibility of ulceration and natural bleeding. The values for each tooth were added up to calculate the individual's total mean GI.

#### Microbiological analysis

To obtain subgingival microbiome samples from periodontal pockets, sterile #15 paper points were placed in the gingival sulcus at four sites: two maxillary teeth (#16 and #21) and two mandibular teeth (#36 and #41) with pocket depth (PD) less than 4 mm for 10 s. They were then placed in sterile 1.5 mL tubes and frozen at − 20 °C until right before an analysis. DNA was dragged from collected #15 paper points using the AccuPrep Universal RNA Extraction Kit (Bioneer, Daejeon, Korea) following the manufacturer's instructions. As shown in Supplementary Table 1, we used three oligonucleotides (forward primer, reverse primer, probe) and OligoMix (YD Global Life Science Co., Ltd., Seongnam, Korea) that react particularly to each bacterium^27^. Also, to prepare reaction samples of polymerase chain reaction (PCR), we combined the following: 9 µL of OligoMix, 10 µL of 2 × probe qPCR mix (Takara Bio Inc., Shiga, Japan), and 1 µL of template DNA. A 96-well plate with PCR reaction samples was placed in a CFX96 Touch Real-Time PCR Detection System (Bio-Rad, Hercules, USA) for DNA amplification. The cycle requirements of PCR were as follows: PCR initial activation step at 95 °C for 30 s, denaturation at 95 °C for 10 s, annealing at 62 °C for 30 s with 40 repeated cycles. The Bio-Rad CFX Manager Software program was used to calculate the cycle threshold (Ct) parameter, and Ct values ​were plotted on a standard curve for each bacterium to figure out the number of copies.

### Statistical analysis

An analysis of frequency was conducted on the demographic characteristics of the participants in this study. SPSS 24.0 for Windows (IBM Corp., Armonk, NY, USA) was used to verify the significance of the measured O'Leary index, PI, GI, and subgingival plaques between the saline group and the *Sambucus williamsii* var. *coreana* extract group performing independent t-test at level 5%. One-way ANOVA was conducted on ‘Baseline’, ‘Treatment’, and ‘After 5 Days’, which are changes according to the application time of gargling. Tukey's test was analyzed as a posteriori test.

### Ethics approval and consent to participate

The study was approved by Institutional Review Board of Silla University (1041449-202008-HR-001, Busan, Korea). Informed consent was obtained from all individual participants included in the study. All methods have been carried out in accordance with the relevant guidelines and regulations.

## Results

### Population characteristics

Table 1 shows the general features of the study subjects. The saline group consisted of 19 females and 13 males, and the *Sambucus williamsii* var*. coreana* group consisted of 20 females and 12 males, so there was no significant difference in gender distribution (*p* > 0.05). The saline group was 36.13 ± 16.48 years old, while the *Sambucus williamsii* var*. coreana* group was 38.06 ± 17.68 years old, so there was no significant difference between the two groups concerning the average age of the subjects (*p* > 0.05)., There were 29 patients without systemic disease and 3 patients with systemic disease in both groups; two had hypertension and one had diabetes. In both groups, 17 were single, and 15 were married, and there was no significant difference between the two groups in systemic disease and marriage (*p* > 0.05).

**Table 1:** Characteristics of the subjects in the saline group and *Sambucus williamsii *var*. coreana* group.

Characteristics	N (%)		
	Saline group (N = 32)	*Sambucus williamsii *var.* coreana *group (N = 32)	*p-*value
Gender*			
Male	13 (40.6)	12 (37.5)	0.424
Female	19 (59.4)	20 (62.5)	
Age^￥^ (mean ± SD)	36.13 ± 16.48	38.06 ± 17.68	0.647
Systemic disease*			
No disease	29 (90.6)	29 (90.6)	0.634
Have a disease	3 (9.4)	3 (9.4)	
Marriage*			
Single	17 (53.1)	17 (53.1)	0.497
Married	15 (46.9)	15 (46.9)	

^￥^*p*-values are determined by independent t-test, *p-values are determined by chi-square test, (*p* < 0.05). Values are means ± standard deviations.

### Clinical indicators related to periodontal disease

Table 2 shows the measured results for the O’Leary index, PI, and GI of the saline gargle group and the *Sambucus williamsii* var. *coreana* extract gargle group. There was no difference concerning the ‘Baseline’ (*p* > 0.05), but there was a significant difference between the two groups concerning ‘Treatment’ and ‘After 5 Days’ (*p* < 0.05). When comparing the two groups concerning periodontal disease-related indexes, such as O'Leary index, PI, and GI, the application of mouthwash containing *Sambucus williamsii* var*. coreana* extract resulted in lower values, indicating that the clinical improvements in the oral environment. In the case of the saline gargle group, all indicators in ‘Baseline’, ‘Treatment’, and ‘After 5 days’ did not show a significant difference (*p* > 0.05). On the other hand, in the *Sambucus williamsii* var*. coreana* extract gargle group, all clinical indicators showed significantly lower values as the application time increased (*p* < 0.05).

**Table 2:** Clinical outcomes observed between two groups.

Variables	Group	Mean ± SD			
		Baseline	Treatment	After 5 days	*p-value
O’Leary index	Saline (N = 32)	50.82 ± 8.85^a^	48.50 ± 9.62^a^	40.29 ± 8.23^a^	0.099
	*Sambucus williamsii *var.* coreana* (N = 32)	50.50 ± 9.50^a^	23.00 ± 7.86^b^	17.10 ± 5.22^b^	**0.000**
	^￥^*p-*value	0.947	**0.000**	**0.000**	
Plaque index (PI)	Saline (N = 32)	1.72 ± 0.41^a^	1.61 ± 0.37^a^	1.46 ± 0.34^a^	0.120
	*Sambucus williamsii* var.* coreana* (N = 32)	1.93 ± 0.33^a^	0.67 ± 0.21^b^	0.44 ± 0.13^b^	**0.000**
	^￥^*p-*value	0.586	**0.000**	**0.000**	
Gingival index (GI)	Saline (N = 32)	1.01 ± 0.21^a^	1.01 ± 0.16^a^	0.96 ± 0.10^a^	0.083
	*Sambucus williamsii* var.* coreana* (N = 32)	1.01 ± 0.22^a^	0.61 ± 0.15^b^	0.32 ± 0.15^c^	**0.000**
	^￥^*p-*value	0.163	**0.000**	**0.000**	

^￥^*p*-values are determined by independent t-test, * *p*-values are determined by one-way ANOVA and different letters (a, b, and c) by the presented statistically significant result of the post hoc Tukey HSD (*P* < 0.05). Values are means ± standard deviations. Significant (bold).

### Distribution of periodontal disease-causing bacteria in the study subjects

Table 3 shows the results of confirming the distribution of bacteria in the oral cavity of the study subjects. Among Gram-positive bacteria, those with *P. micra* had the highest proportion in the oral, and the average of bacteria was also high. Among Gram-negative bacteria, those with *F. nocleatum* had the highest proportion in the oral, and the average of bacteria was also high.

**Table 3:** Distribution of periodontal disease-causing bacteria in the study subjects (N = 64).

Variables		N (%)	Mean ± SD
Gram-positive	*Parvimonas micra*	64 (100.0)	1355.01 ± 1505.33
	*Staphylococcus aureus*	8 (12.50)	10.28 ± 22.86
	*Eubacterium nodatum*	25 (39.06)	23.65 ± 27.16
Gram-negative	*Porphyromonas gingivalis*	36 (56.25)	126.65 ± 112.66
	*Treponema denticola*	18 (28.13)	5.48 ± 5.73
	*Fusobacterium nocleatum*	64 (100.0)	322,748.88 ± 243,220.17
	*Prevotella intermedia*	25 (39.06)	10,562.88 ± 16,832.79
	*Prevotella nigrescens*	58 (90.63)	1011.47 ± 956.47
	*Eikenella corrodens*	25 (39.06)	115.13 ± 126.89
	*Campylobacter rectus*	18 (28.13)	123.5 ± 95.89

### Gram-positive bacteria in subgingival plaques

Gram-positive bacterial data in subgingival plaques was detected as three types of bacteria: *P. micra, Staphylococcus aureus (S. aureus), and Eubacterium nodatum (E. nodatum)*. In addition, the effect difference between the two groups was verified according to the maxillary and mandible (Table 4). When comparing the saline gargle group and the *Sambucus williamsii* var. *coreana* extract gargle group against *P. micra*, there was a significant difference only in the mandible at the time of ‘Treatment’ and ‘After 5 days’ (*p* < 0.05). There was a significant difference in ‘After 5 days’ in the maxilla (*p* < 0.05) regarding *S. aureus*; it was not detected in both groups in the mandible. There was no significant difference between the two groups for *E. nodatum* in the oral cavity (*p* > 0.05). Also, based on the ‘Baseline’, there was a significant difference from ‘Treatment’ to ‘After 5 days’ in the mandible of the *Sambucus williamsii coreana* var. extract gargle group in the case of *P. micra* (*p* < 0.05). As for *E. nodatum*, there was a significant difference in both groups in the maxilla (*p* < 0.05).

**Table 4:** Gram-positive bacterial measurements in subgingival plaque (3 types).

Variables	Group	Mean ± SD			
		Baseline	Treatment	After 5 days	*p-value
***Parvimonas micra***					
Maxilla	Saline (N = 32)	921.43 ± 654.03^a^	879.34 ± 1752.96^a^	112.88 ± 104.13^a^	0.331
	*Sambucus williamsii* var.* coreana* (N = 32)	957.03 ± 1666.78^a^	88.30 ± 116.49^a^	93.12 ± 56.66^a^	0.159
	^￥^*p-*value	0.957	0.222	0.674	
Mandible	Saline (N = 32)	1708.80 ± 2011.47^a^	1646.82 ± 1007.24^a^	1291.93 ± 371.47^a^	0.824
	*Sambucus williamsii* var.* coreana* (N = 32)	1832.78 ± 1689.04^a^	29.38 ± 26.88^b^	357.00 ± 336.00^b^	**0.007**
	^￥^*p-*value	0.901	**0.000**	**0.000**	
***Staphylococcus aureus***					
Maxilla	Saline (N = 32)	18.63 ± 21.43^a^	18.13 ± 20.99^a^	11.13 ± 13.91^a^	0.697
	*Sambucus williamsii* var.* coreana* (N = 32)	22.50 ± 24.29^a^	16.30 ± 19.58^a^	0.00 ± 0.00^a^	0.065
	^￥^*p-*value	0.745	0.862	**0.037**	
Mandible	Saline (N = 32)	0.00 ± 0.00	0.00 ± 0.00	0.00 ± 0.00	–
	*Sambucus williamsii* var.* coreana* (N = 32)	0.00 ± 0.00	0.00 ± 0.00	0.00 ± 0.00	**–**
	^￥^*p-*value	–	–	–	
***Eubacterium nodatum***					
Maxilla	Saline (N = 32)	50.27 ± 50.97^a^	13.70 ± 19.27^a,b^	9.37 ± 14.24^b^	**0.032**
	*Sambucus williamsii* var.* coreana* (N = 32)	25.60 ± 30.15^a^	0.67 ± 0.80^a,b^	5.80 ± 7.87^b^	**0.022**
	^￥^*p-*value	0.260	0.074	0.551	
Mandible	Saline (N = 32)	9.21 ± 18.87^a^	68.20 ± 104.05^a^	4.72 ± 4.20^a^	0.770
	*Sambucus williamsii* var.* coreana* (N = 32)	9.50 ± 8.64^a^	3.30 ± 2.22^a^	3.12 ± 3.81^a^	0.055
	^￥^*p-*value	0.970	0.178	0.478	

^￥^*p*-values are determined by independent t-test, *p-values are determined by one-way ANOVA and different letters (a and b) by the presented statistically significant result of the post hoc Tukey HSD (*P* < 0.05). Values are means ± standard deviations. Significant (bold).

### Gram-negative bacteria in subgingival plaques

The following seven types of bacteria were detected as gram-negative bacteria in the oral cavity: *P. gingivalis*, *T. denticola*, *F. nocleatum*, *P. intermedia*, *P. nigrescens*, *Eikenella corrodens (E. corrodens)*, and *Campylobacter rectus (C. rectus)*. In addition, the effect difference between the two groups was verified according to the maxillary and mandible (Table 5). When comparing the saline gargle group and the *Sambucus williamsii* var. *coreana* extract gargle group, *P. gingivalis* showed a significant difference at ‘Treatment’ and ‘After 5 days’ in maxilla and mandible (*p* < 0.05). *T. denticola* was detected only in the mandible, showing a significant difference between the two groups at ‘Treatment’ and ‘After 5 days’ (*p* < 0.05). *F. nocleatum* differed between groups only in the maxilla ‘After 5 days’ (*p* < 0.05). As for *P. intermedia* showed a significant difference between the groups at ‘Treatment’ and ‘After 5 days’ only in the mandible (*p* < 0.05). In the case of *P. nigrescens*, there was a significant difference between the two groups ‘After 5 days’ in both maxilla and mandible (*p* < 0.05). *E. corrodens* was not significantly different between the two groups in the maxilla and mandible. *C. rectus* showed a difference between the two groups at ‘Treatment’ and ‘After 5 days’ in the case of maxilla but showed a significant difference only at ‘Treatment’ in the mandible (*p* < 0.05). According to the application time, the changes were not significantly different in the saline gargle group from ‘Baseline’ to ‘After 5 days’ except for *F. nocleatum* in the maxilla and *E. corrodens* in the mandible, among the seven types of gram-negative bacteria (*p* > 0.05). On the other hand, in the *Sambucus williamsii* var. *coreana* extract gargle group, all bacteria in the maxilla and mandible were significantly reduced according to the application time, except for *F. nocleatum* in the mandible; *P. intermedia* and *P. nigrescens* of the maxilla. In particular, based on ‘Baseline’, *P. gingivalis* and *C. rectus* showed a marked differences from ‘Treatment’ to ‘After 5 days’ in the maxilla and mandible, verifying that gram-negative bacteria decreased clearly. From ‘Treatment’ to ‘After 5 days’, *T. denticola, P. intermedia*, and *E. corrodens* were reduced in the mandible only, and *T. denticola* was not even detected at ‘Treatment’ and ‘After 5 days’.

**Table 5:** Gram-negative bacterial measurements in subgingival plaque (7 types).

Variables	Group	Mean ± SD			
		Baseline	Treatment	After 5 days	*p-value
***Porphyromonas gingivalis***					
Maxilla	Saline (N = 32)	109.30 ± 133.68^a^	47.67 ± 24.08^a^	50.93 ± 38.28^a^	0.262
	*Sambucus williamsii* var.* coreana* (N = 32)	57.67 ± 53.35^a^	7.93 ± 10.45^b^	6.15 ± 6.89^b^	**0.005**
	^￥^*p-*value	0.352	**0.000**	**0.004**	
Mandible	Saline (N = 32)	171.10 ± 140.72^a^	206.47 ± 146.23^a^	238.55 ± 151.38^a^	0.681
	*Sambucus williamsii* var.* coreana* (N = 32)	168.53 ± 122.87^a^	10.33 ± 6.26^b^	10.60 ± 7.12^b^	**0.000**
	^￥^*p-*value	0.970	**0.001**	**0.000**	
***Treponema denticola***					
Maxilla	Saline (N = 32)	0.00 ± 0.00	0.00 ± 0.00	0.00 ± 0.00	–
	*Sambucus williamsii* var.* coreana* (N = 32)	0.00 ± 0.00	0.00 ± 0.00	0.00 ± 0.00	–
	^￥^*p-*value	–	–	–	
Mandible	Saline (N = 32)	10.50 ± 12.29^a^	6.40 ± 7.22^a^	8.07 ± 9.04^a^	0.716
	*Sambucus williamsii* var.* coreana* (N = 32)	11.43 ± 10.62^a^	0.00 ± 0.00^b^	0.00 ± 0.00^b^	**0.003**
	^￥^*p-*value	0.876	**0.022**	**0.021**	
***Fusobacterium nocleatum***					
Maxilla	Saline (N = 32)	412,231.93 ± 237,792.45^a^	316,335.28 ± 249,760.27^a^	33,533.67 ± 24,271.80^b^	**0.004**
	*Sambucus williamsii* var.* coreana* (N = 32)	333,965.80 ± 322,845.65^a^	104,002.86 ± 144,013.44^a,b^	11,462.04 ± 4562.88^b^	**0.016**
	^￥^*p-*value	0.608	0.069	**0.038**	
Mandible	Saline (N = 32)	279,433.90 ± 210,103.94^a^	273,512.70 ± 212,041.39^a^	158,732.78 ± 126,279.75^a^	0.409
	*Sambucus williamsii* var.* coreana* (N = 32)	265,363.90 ± 202,138.62^a^	129,837.11 ± 145,893.87^a^	95,250.00 ± 92,088.18^a^	0.110
	^￥^*p-*value	0.896	0.139	0.315	
***Prevotella intermedia***					
Maxilla	Saline (N = 32)	390.59 ± 4783.59^a^	3941.66 ± 6515.62^a^	471.63 ± 625.49^a^	0.300
	*Sambucus williamsii* var.* coreana* (N = 32)	40,268.10 ± 61,402.20^a^	1720.47 ± 3149.98^a^	173.54 ± 208.53^a^	0.057
	^￥^*p-*value	0.117	0.406	0.248	
Mandible	Saline (N = 32)	774.43 ± 617.20^a^	413.86 ± 362.89^a^	275.15 ± 269.36^a^	0.108
	*Sambucus williamsii* var.* coreana* (N = 32)	818.38 ± 528.18^a^	81.40 ± 114.22^b^	54.37 ± 50.66^b^	**0.000**
	^￥^*p-*value	0.886	**0.027**	**0.049**	
***Prevotella nigrescens***					
Maxilla	Saline (N = 32)	1021.38 ± 909.20^a^	754.78 ± 342.72^a^	801.04 ± 279.63^a^	0.679
	*Sambucus williamsii* var.* coreana* (N = 32)	1093.63 ± 1158.02^a^	1064.70 ± 1542.44^a^	293.89 ± 275.80^a^	0.338
	^￥^*p-*value	0.901	0.596	**0.002**	
Mandible	Saline (N = 32)	971.58 ± 788.81^a^	851.69 ± 924.14^a^	500.36 ± 232.09^a^	0.482
	*Sambucus williamsii* var.* coreana* (N = 32)	959.30 ± 969.86^a^	219.78 ± 187.43^a,b^	97.29 ± 98.77^b^	**0.025**
	^￥^*p-*value	0.980	0.088	**0.000**	
***Eikenella corrodens***					
Maxilla	Saline (N = 32)	24.73 ± 19.52^a^	21.40 ± 16.45^a^	17.96 ± 15.61^a^	0.759
	*Sambucus williamsii* var.* coreana* (N = 32)	53.80 ± 52.30^a^	20.40 ± 21.15^a,b^	8.30 ± 10.74^b^	**0.033**
	^￥^*p-*value	0.162	0.919	0.186	
Mandible	Saline (N = 32)	184.89 ± 179.89^a^	68.20 ± 104.05^a,b^	7.73 ± 10.71^b^	**0.019**
	*Sambucus williamsii* var.* coreana* (N = 32)	197.11 ± 255.86^a^	23.40 ± 35.70^b^	15.56 ± 15.59^b^	**0.040**
	^￥^*p-*value	0.919	0.272	0.280	
***Campylobacter rectus***					
Maxilla	Saline (N = 32)	71.23 ± 74.59^a^	57.40 ± 56.93^a^	29.40 ± 31.02^a^	0.356
	*Sambucus williamsii* var. *coreana* (N = 32)	64.97 ± 60.09^a^	4.00 ± 4.55^b^	2.78 ± 2.67^b^	**0.001**
	^￥^*p-*value	0.860	**0.016**	**0.026**	
Mandible	Saline (N = 32)	167.83 ± 57.83^a^	115.70 ± 140.49^a^	111.97 ± 137.50^a^	0.693
	*Sambucus williamsii* var. *coreana* (N = 32)	189.97 ± 191.03^a^	6.22 ± 8.97^b^	38.40 ± 73.79^b^	**0.010**
	^￥^*p-*value	0.826	**0.042**	0.210	

^￥^*p*-values are determined by independent t-test, *p-values are determined by one-way ANOVA and different letters (a and b) by the presented statistically significant result of the post hoc Tukey HSD (*P* < 0.05). Values are means ± standard deviations. Significant (bold).

## Discussion

About 700 types of microorganisms live in the oral cavity; because the nutrients necessary for the growth of microorganisms are continuous, the bacteria proliferate to form dental plaques that cause oral bacterial diseases^28^. Therefore, the best way to prevent this is to remove the dental plaque, representatively using Tetracyclines, Metrodinidazole, Penicillin, Clindamycin, and Ciprofloxacin out of the various types of antibacterial agents^29^. These chemical antibacterial agents inhibit bone loss and are effective for periodontitis^30^, but their use is limited due to side effects such as the generation of resistant bacteria and mycosis^31^.

Therefore, this study verified the O’Leary index, PI, and GI as periodontal-related indicators; and antibacterial effects on pathogens causing periodontal diseases to develop a natural mouthwash using *Sambucus williamsii* var. *coreana* extract, which has a natural antibiotic effect.

The high baseline values of the PI, GI, and bacteria seem to come out due to the lack of oral care before applying mouthwash. After measuring the baselines, two examiners (dental hygienists) also educated the subjects about how to use mouthwash and how often and how to brush teeth using the provided toothpaste and toothbrush. As a result of this study, O'Leary index, PI, and GI all showed marked decreases from ‘Treatment’ to ‘After 5 days’ compared to the saline gargle group when applying mouthwash containing *Sambucus williamsii* var*. coreana* extract. Also, as the application time of the mouthwash containing *Sambucus williamsii* var*. coreana* extract increased, the values of periodontal-related indicators were effectively lowered, verifying that there was an effect of improving the oral environment.

The immediate improvement in GI in this study is shown as a statistical result by a small number of subjects with a large number of subjects with healthy gingiva and a large change in GI improvement.

According to Cai et al.’s study^32^, herb mouthwash has mentioned potential benefits in plaque and inflammation control as a supplement to routine oral hygiene in patients with gingivitis, and in particular, Balappanavar et al.’s study^33^ and Meena Priya et al.’s study^34^ showed a greater reduction in gingivitis after intervention.

According to a previous study, *Sambucus williamsii* var*. coreana* extract had antioxidant and anti-inflammatory effects and had no cytotoxicity^35^. Therefore, applying it in the oral cavity as a mouthwash is considered appropriate to suppress and prevent periodontal disease.

*P. micra*, which is known as a bacterium associated with periodontal disease, is a gram-positive, non-motile, anaerobic coccus^36^ that is more common in patients with severe or moderate periodontitis than those with gingivitis and mild periodontitis^37^. In this study, a mouthwash containing *Sambucus williamsii* var*. coreana* extract was effective in the mandible; the antibacterial effect appeared immediately after application and continued until ‘After 5 days’. *P. micra* is known to act as a core pathogen in the development of the periodontal disease^38^; signaling molecules derived from *P. micra* stimulate the expression of the *P. gingivalis* proteolytic gingipain enzyme thirteen times more to enhance the growth and toxicity of Porphyromonas gingivalis, a major periodontal pathogen^39^. *P. gingivalis*, a gram-negative anaerobic bacterium, is associated with periodontal destruction and increasing risk of disease recurrence^40^; it is the foremost periopathogen for periodontal disease progression^41^. After applying the mouthwash containing *Sambucus williamsii* var*. coreana* extract to *P. gingivalis*, it decreased sharply in the maxilla and the mandible compared to the saline gargle group. The mouthwash containing *Sambucus williamsii* var. *coreana* extract was verified to be very effective against *P. gingivalis* as it decreased continuously right after applying the mouthwash. Therefore, a mouthwash using *Sambucus williamsii* var*. coreana* extract can inhibit and block the growth of core pathogens of periodontal disease. *P. gingivalis* is also considered the major pathogen because of its ability to change the normal microflora into a dysbiotic community^42^. Among dysbiotic species, the increase in *P. gingivalis*, a major periodontal disease-related pathogen, is accompanied by various bacteria, including *P. intermedia* and *F. nucleatum*^43^. Since the antibacterial effect was shown by a marked reduction of *P. gingivalis* in this study, mouthwash containing *Sambucus williamsii* var. *coreana* extract will play a huge role in reducing various anaerobic bacteria. *P. intermedia*, the anaerobic black bacteria, is one of the powerful causative agents of adult-type periodontitis and various types of periodontal disease^44^. In addition, since the expression frequency of *P. nigrenscens* was reported to be more than double that of *P. intermedia* in the inflammatory site of patients with periodontal disease^45^, dealing with *P. nigrenscens* is also necessary. Compared with the saline gargle group, *P. intermedia* effectively decreased in the mandible immediately after applying a mouthwash containing *Sambucus williamsii* var*. coreana* extract. There was no effect on *P. nigrenscens* immediately after using a mouthwash containing *Sambucus williamsii* var*. coreana* extract, but it became effective in both the maxilla and mandible after using the mouthwash for five days. Both *P. intermedia* and *P. nigrenscens* more effectively decreased over time in the mandible as applying a mouthwash containing *Sambucus williamsii* var. *coreana* extract. As a result, a mouthwash containing *Sambucus williamsii* var*. coreana* extract reduced *P. gingivalis* immediately after applying a mouthwash. *P. nigrenscens* and *P. intermedia* also decreased as the application time increased. *F. nocleatum,* one of the oral microflora and a causative agent of periodontitis, is also related to preterm birth, colorectal cancer, inflammatory bowel disease, respiratory tract infections, cardiovascular disease, rheumatoid arthritis, and Alzheimer’s disease^46^. *T. denticola*, a bacterium that causes progressive periodontitis, is associated with losing attached gingiva and bleeding when measuring the periodontal pocket and its depth^3^. In the results of this study, *F. nocleatum* was reduced compared to the saline gargle group at ‘After 5 days’ in the maxilla upon using a mouthwash containing *Sambucus williamsii* var*. coreana* extract. On the other hand, in the case of *T. denticola*, the mouthwash containing *Sambucus williamsii* var*. coreana* extract was effective until ‘After 5 days’ as the bacteria completely disappeared from ‘Treatment’ in the mandible. In addition, *E. corrodens* is a bacterium that causes acute periodontitis and promotes complex infection. *C. rectus* is a bacterium involved in root canal infection and periodontal disease^47^. Both bacteria appear in patients with severe periodontal disease. When comparing the saline gargle group and the *Sambucus williamsii* var*. coreana* extract gargle group, there was no significant difference between the two groups regarding *E. corrodens*. In the case of the saline gargle group, there was a significant difference in the mandible at 'After 5 days,' the increased application time; if the application time increases, the reduction of acute periodontitis-causing bacteria can occur even with a saline. The number of *C. rectus* decreased in *Sambucus williamsii* var*. coreana* extract gargle group showing the antibacterial effect. From immediate applying a mouthwash containing *Sambucus williamsii* var. *coreana* extract, bacteria showed differences continuously in both the maxilla and the mandible. According to these oral clinical results, we verified *Sambucus williamsii* var*. coreana* extract as an excellent natural substance with antibacterial effects for improving periodontal disease.

As periodontal disease progresses, bacterial species diversify^48^, so continuous management of anaerobic bacteria related to periodontal disease is required. Based on the study results, a mouthwash containing *Sambucus williamsii* var*. coreana* extract reduced bacteria in the oral cavity such as *P. gingivalis, C. rectus, T. denticola, P. intermedia,* and *E. corrodens* immediately after application. As the application time of a mouthwash containing *Sambucus williamsii* var*. coreana* extract extended, a more marked antibacterial effect appeared, which means it is excellent in improving periodontal disease. According to the results of this study, mouthwash was more effective in the mandible; it seems that the teeth and periodontal tissues of the mandible were in contact with and stored more in the mouthwash when keeping the mouthwash in the mouth. Therefore, to maximize the effect of mouthwash, it will be more effective to gargle mouthwash enough to cover the entire oral cavity rather than simply holding it.

The limitation of this study is that a mouthwash containing *Sambucus willamsii* var. *coreana* extractwas applied only for five days to verify its effects. For this reason, it can be considered that the use of mouthwash for a short period of time alone has the effect of improving gingivitis. However, further research is needed through clinical efficacy verification to confirm the improvement of periodontitis by expanding the subjects to periodontal disease patients and lengthening the period of mouthwash use. In addition, more systemic disease variables will be corrected in order to secure the homogeneity of study subjects for future research, and the reliability of the results of clinical trials will be secured through 1:1 propensity score matching.

Based on this study verifying the practicality and development of oral health care products using *Sambucus williamsii* var*. coreana* extract, a mouthwash containing *Sambucus williamsii* var*. coreana* extract can inhibit and prevent periodontal disease. As a natural ingredient with sufficiently excellent effects, *Sambucus williamsii* var*. coreana* extract can be used for oral health by improving periodontal disease.

## Conclusion

A mouthwash containing *Sambucus williamsii* var. *coreana* extract affects oral hygiene by reducing the O’Leary index, PI, and GI. It also has a potential advantage in inflammation control by reducing periodontal disease-causing bacteria, so alternative oral care products as the adjuvant treatment of periodontal disease can be possible. In addition, medicine using *Sambucus williamsii* var*. coreana* extract rather than chemicals can be a promising field for treating gingivitis.

## Supplementary Information

Supplementary Information 1.

Supplementary Information 2.

Supplementary Information 3.

## Data Availability

The data sets generated and/or analyzed during the current study are not publicly available for reasons of personal and organizational integrity but are available from the corresponding author on reasonable request.

## Abbreviations

PI: Plaque index
GI: Gingival index
*P. micra*: *Parvimonas micra*
*S. aureus*: *Staphylococcus aureus*
*E. nodatum*: *Eubacterium nodatum*
*P. gingivalis*: *Parvimonas micra*
*T. denticola*: *Treponema denticola*
*F. nucleatum*: *Fusobacterium nucleatum*
*P. intermedia*: *Prevotella intermedia*
*P. nigrescens*: *Prevotella nigrescens*
*E. corrodens*: *Eikenella corrodens*
*C. rectus*: *Campylobacter rectus*

## References

[1] Kato A, Imai K, Ochiai K, Ogata Y (2013). Higher prevalence of Epstein-Barr virus DNA in deeper periodontal pockets of chronic periodontitis in Japanese patients. PLoS ONE.

[2] Könönen E, Gursoy M, Gursoy UK (2019). Periodontitis: A multifaceted disease of tooth-supporting tissues. J. Clin. Med..

[3] Boutaga K, van Winkelhoff AJ, Vandenbroucke-Grauls CM, Savelkoul PH (2013). Comparison of real-time PCR and culture for detection of Porphyromonas gingivalis in subgingival plaque samples. J. Clin. Microbiol..

[4] Borrell LN, Papapanou PN (2005). Analytical epidemiology of periodontitis. J. Clin. Periodontol..

[5] Garlet GP (2010). Destructive and protective roles of cytokines in periodontitis: A re-appraisal from host defense and tissue destruction viewpoints. J. Dent. Res..

[6] Butler LD (1989). Interleukin 1-induced pathophysiology: Induction of cytokines, development of histopathologic changes, and immunopharmacologic intervention. Clin. Immunol. Immunopathol..

[7] Birkedal-Hansen H (1993). Role of cytokines and inflammatory mediators in tissue destruction. J. Periodont. Res..

[8] Yamamoto M, Fujihashi K, Hiroi T, McGhee JR, Van Dyke TE, Kiyono H (1997). Molecular and cellular mechanisms for periodontal diseases: Role of Th1 and Th2 type cytokines in induction of mucosal inflammation. J. Periodont. Res..

[9] Ciancio SG (1989). Agents for the management of plaque and gingivitis. J. Am. Coll. Dent..

[10] To KK (2020). Consistent detection of 2019 novel coronavirus in saliva. Clin. Infect. Dis..

[11] In the era of COVID-19, wise oral care. *Ministry of Health and Welfare.* (2021).

[12] Lis-Balchin M (1977). Essential oils and aromatherapy: Their modern role in healing. J. R. Soc. Health..

[13] An JY, Lee SS, Kang HY (2004). Biological activities of essential oil from chamaecyparis obtusa. J. Soc. Cosmet. Scientists Korea.

[14] Palombo EA (2011). Traditional medicinal plant extracts and natural products with activity against oral bacteria: Potential application in the prevention and treatment of oral diseases. Evid. Based Complement. Alternat. Med..

[15] Seyed Hashemi M (2019). The efficacy of asafoetida (*Ferula*
*assa-foetida* oleo-gum resin) versus chlorhexidine gluconate mouthwash on dental plaque and gingivitis: A randomized double-blind controlled trial. Eur. J. Integr. Med..

[16] Rashidi Maybodi F, Vaziri F, Ghanbarnezhad S, Herandi V (2022). The effect of aqueous extract of *Crocus sativus* L. (Saffron) on periodontal indices of patients with generalized periodontitis. Tradit. integr. Med..

[17] Villinski JR (2014). Pyrano-isoflavans from Glycyrrhiza uralensis with antibacterial activity against streptococcusmutans and porphyromonasgingivalis. J. Nat. Prod..

[18] Jung GO, Seo SY, Yoon SU (2020). Anti-microbial activity of bamboo extract against oral microbes. J. Koon..

[19] Loe H (1973). Does chlorhexidine have a place in the prophylaxis of dental disease?. J. Periodontal Res. Suppl..

[20] Haffajee AD, Socransky SS (2000). Microbial etiological agents of destructive periodontal diseases. Periodontol..

[21] Lee SC, Kang HM, Yu SB, Choi SH (2020). Vegetation characteristics and changes of evergreen broad-leaved forest in the cheomchalsan(Mt.) at Jindo(Island). Korean J. Ecol..

[22] Yang XJ, Wong MS, Wang NL, Chan SC, Yao XS (2007). Lignans from the stems of *Sambucus williamsii* and their effects on osteoblastic UMR106 cells. J. Asian Nat. Prod. Res..

[23] Nam SH (2019). Antibacterial effect of Sambucus Williamsii Var. Coreana NAKAI (S. Williamsii) against Enterococcus faecalis (E. faecalis) for the Traditional Treatment of Oral Diseases. Indian J. Public Health Res..

[24] Lang NP, Tonetti MS (2003). Periodontal risk assessment (PRA) for patients in supportive periodontal therapy (SPT). Oral Health Prev. Dent..

[25] Loe H, Silness J (1963). Periodontal disease in pregnancy. I. Prevalence and severity. Acta Odontol. Scand..

[26] Silness J, Loe H (1964). Periodontal disease in pregnancy. II. Correlation between oral hygiene and periodontal condition. Acta Odontol. Scand..

[27] Lee, J.W., Kim, M.B. & inventors YD Global Life Science Company patentee. Composition and detection method for simultaneous detection of multiple oral disease-causing bacteria using multiplex real-time PCR. KR Patent 10–1706070.

[28] Mashimo PA, Yamamoto Y, Slots J, Evans RT, Genco RJ (1981). In vitro evaluation of antibiotics in the treatment of periodontal dissease. Pharmacol. Ther. Dent..

[29] Slots J, Rams TE (1990). Antibiotics and periodontal therapy: Advantages and disadvanyages. J. Clin. Periodontol..

[30] Botelho MA (2007). Antimicrobial activity of the essential oil from Lippia sidoides, carvacrol and thymol against oral pathogens. Braz. J. Med. Biol. Res..

[31] Jin HJ, Kim EK, An SY, Im SU, Song KB, Choi YH (2013). Relationship between periodontal status and chronic obstructive pulmonary disease. J. Korean Acad. Oral..

[32] Cai H, Chen J, Perera NK, Liang X (2020). Effects of herbal mouthwashes on plaque and inflammation control for patients with gingivitis: a systematic review and meta-analysis of randomised controlled trials. Evid. Based Complement Alternat. Med..

[33] Balappanavar AY, Sardana V, Singh M (2013). Comparison of the effectiveness of 0.5% tea, 2% neem, and 0.2% chlorhexidine mouthwashes on oral health: a randomized control trial. Indian J. Dent. Res..

[34] Meena Priya B, Anitha V, Shanmugam M, Ashwath B, Sylva SD, Vigneshwari SK (2015). Efficacy of chlorhexidine and green tea mouthwashes in the management of dental plaque-induced gingivitis: A comparative clinical study. Contemp. Clin. Dent..

[35] Kim KT (2013). Antioxidant activity and protective effects of extracts from *Sambucus williamsii var. **coreana* on *t*-BHP induced oxidative stress in chang cells. J. Soc. Korean Med. Diagn..

[36] Rams TE, van Winkelhoff AJ (2017). Introduction to clinical microbiology for the general dentist. Dent. Clin..

[37] Curtis MA, Diaz PI, Van Dyke TE (2020). The role of the microbiota in periodontal disease. Periodontol..

[38] Al-Hebshi NN, Shuga-Aldin HM, Al-Sharabi AK, Ghandour I (2014). Subgingival periodontal pathogens associated with chronic periodontitis in Yemenis. BMC Oral Health.

[39] Neilands J, Davies JR, Bikker FJ, Svensäter G (2019). *Parvimonas micra* stimulates expression of gingipains from *Porphyromonas gingivalis* in multi-species communities. Anaerobe.

[40] Socransky SS, Smith C, Haffajee AD (2002). Subgingival microbial profiles in refractory periodontal disease. J. Clin. Periodontol..

[41] Socransky SS, Haffajee AD (2005). Periodontal microbial ecology. Periodontol.

[42] Romacho T, Villalobos LA, Cercas E, Carraro R, Sánchez-Ferrer CF, Peiró C (2013). Visfatin as a novel mediator released by inflamed human endothelial cells. PLoS ONE.

[43] Schwenzer A (2017). Association of distinct fine specificities of anti-citrullinated peptide antibodies with elevated immune responses to Prevotella intermedia in a subgroup of patients with rheumatoid arthritis and periodontitis. Arthritis Rheumatol..

[44] Wennstrom JL, Dahlen G, Svensson J, Nyman S (1987). Actinobacillus actinomycetemcomitans, Bacteroides gingivalis and Bacteroides intermedius: Predictors of attachment loss?. Oral Microbiol. Immunol..

[45] Teanpaisan R, Douglas CWI, Walsh TF (1995). Characterization of black-pigmented anaerobes isolated from diseased and healthy periodontal sites. J. Periodont. Res..

[46] Han YW (2015). Fusobacterium nucleatum: A commensal-turned pathogen. Curr. Opin. Microbiol..

[47] Socransky S, Haffajee A, Cugini M, Smith C, Kent R (1998). Microbial complexes in subgingival plaque. J. Clin. Periodontol..

[48] Simon DT, Rudney JD (1999). Improved multiplex PCR using conserved and species-specific 16S rRNA gene primers for simultaneous detection of actinobacillus actinomycetemcomitans, Bacteroides forsythus, and Porphyromonas gingivalis. J. Clin. Microbiol..

