# Supplementary figures and images for: A randomized, placebo-controlled clinical trial evaluating of a mouthwash containing Sambucus williamsii var. coreana extract for prevention of gingivitits
Source: Sci Rep. 2022 Jul 18;12:11250. doi: 10.1038/s41598-022-15445-7 (PMC9293903; doi:10.1038/s41598-022-15445-7)

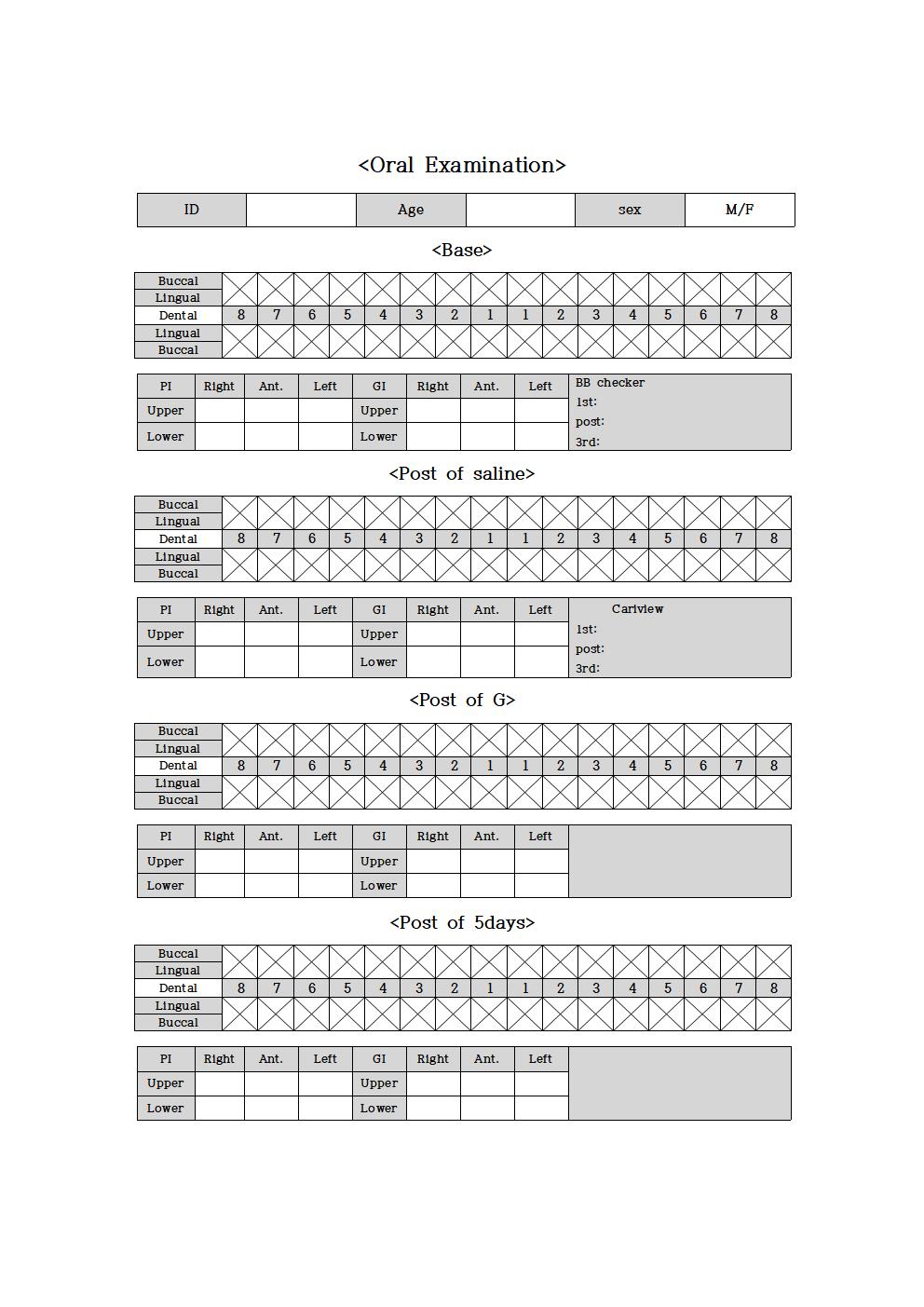

Supplement: Supplementary file 1 — Supplementary Information 1. [file 41598_2022_15445_MOESM1_ESM.jpg]

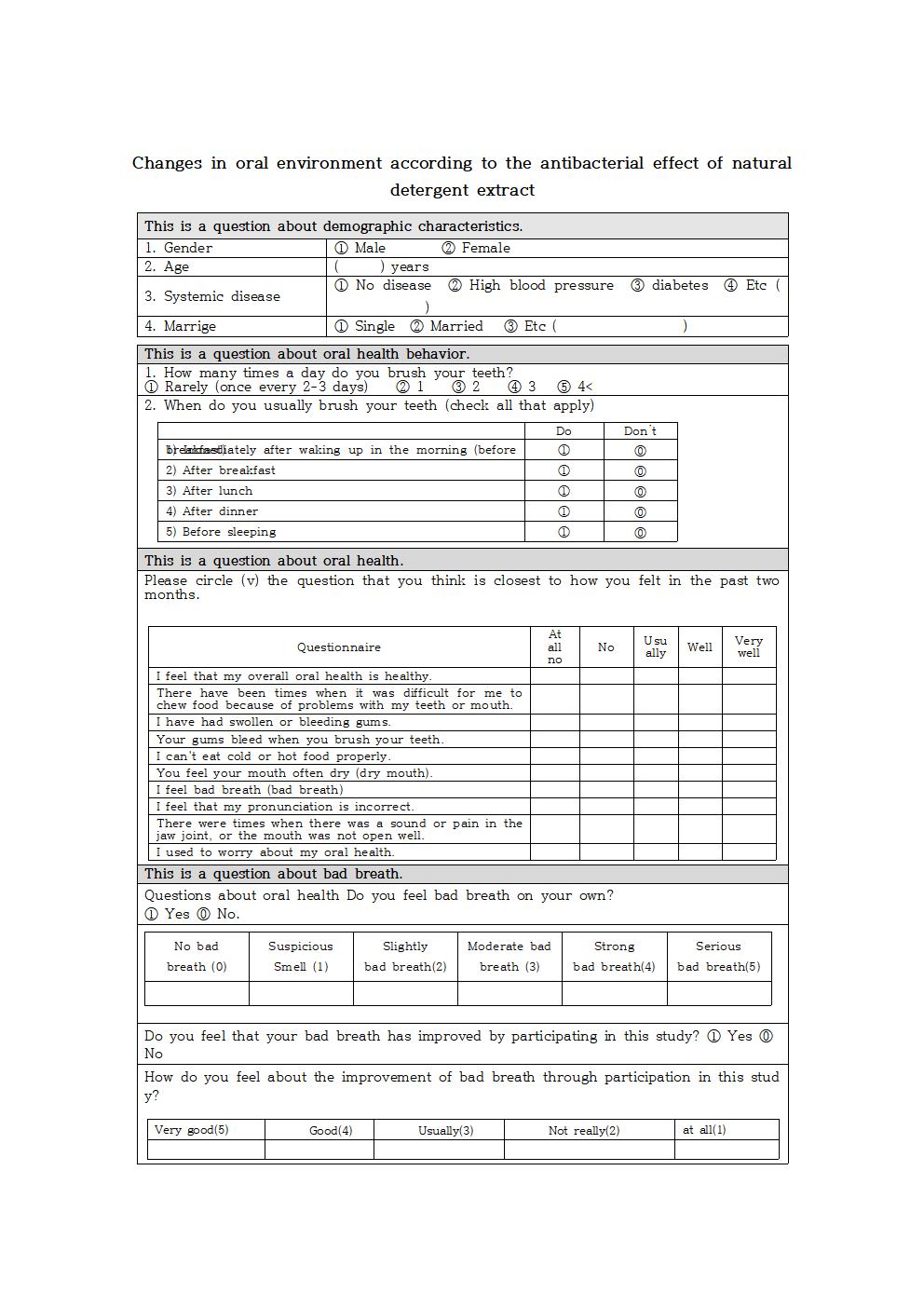

Supplement: Supplementary file 2 — Supplementary Information 2. [file 41598_2022_15445_MOESM2_ESM.jpg]
