# Supplementary material for: A randomized, placebo-controlled clinical trial evaluating of a mouthwash containing Sambucus williamsii var. coreana extract for prevention of gingivitits
Source: Sci Rep. 2022 Jul 18;12:11250. doi: 10.1038/s41598-022-15445-7 (PMC9293903; doi:10.1038/s41598-022-15445-7)
Supplement: Supplementary file 3 — Supplementary Information 3. [file 41598_2022_15445_MOESM3_ESM.docx]

| Supplementary table 1. Primers and probes used in the real-time PCR assays | | | |
| --- | --- | --- | --- |
| Bacteria | Target genes | Primers/Probe sets | Amplicon size (bp) |
| *Parvimonas micra* | 16S ribosomal RNA gene | 5′-GAGGAATACCGGTGGCGAAG-3’  5′-GGCACCGAGATTTGACTCCC-3’  5′-FAM-GGTACGAAAGCGTGGGGAGCA-BHQ1–3’ | 148 |
| *Staphylococcus aureus* | clumping factor A (clfA) gene | 5′-GCGCAAGTAACGAAAGCAAAA-3’  5′-GATTTTGCGCCACACTCGTT-3’  5′-FAM-TGCTGCACCTAAAACAGACGACACA-BHQ1–3’ | 132 |
| *Eubacterium nodatum* | hypothetical protein | 5′-TGCTTGCCGGTGACTTAGGA-3’  5′-AAACCGGGCTCAACAACCAT-3’  5′-Texas Red-TTGAGGAGCCGGTGACTTTGG-BHQ2–3’ | 130 |
| *Porphyromonas gingivalis* | hemagglutinin (phg) gene | 5′-ACACGGTGTATCGTGACGGC-3’  5′-GCCGGCTGCGTACTTAACCT-3’  5′-HEX-CGACCTACCGCGATGCAGGA-BHQ1–3’ | 119 |
| *Treponema denticola* | oligopeptidase B (opdB) gene | 5′-AGAAAGGCTTTGGGCGACAG-3’  5′-GCTGGAGCCGTAGCTTCCAT-3’  5′-Cy5-CGGGTCCTCACCCGCTCTTC-BHQ2–3’ | 127 |
| *Fusobacterium nucleatum* | 16S ribosomal RNA gene | 5′-GGCTGTCGTCAGCTCGTGTC-3’  5′-CTCATCGCAGGCAGTATCGC-3’  5′-FAM-AACGAGCGCAACCCCTTTCG-BHQ1–3’ | 114 |
| *Prevotella intermedia* | hemagglutinin (phg) gene | 5′-CACACGCTGGCGAAACCTAC-3’  5′-CACGTGGCGTTGCTTCTTTC-3’  5′-HEX-CCGAAGATGCGCCGTTGAAC-BHQ1–3’ | 143 |
| *Prevotella nigrescens* | gyrase subunit B (gyrB) gene | 5′-AGCAAGCTGTAGGCGAGGCT-3’  5′-GCTGAACACTTTCGCGTGCT-3’  5′-Texas Red-GCTCGTATTGCAGCCCGCAA-BHQ2–3’ | 132 |
| *Eikenella corrodens* | proline iminopeptidase (pip) gene | 5′-GCCAACTGCTGCTGGAAGTG-3’  5′-GCCGCTGATTTCGGAGAGTT-3’  5′-HEX- ACAGCCATCGGCACAGGCAT-BHQ1–3’ | 110 |
| *Campylobacter rectus* | groEL gene | 5′-AAATTTAAGCGGCGACGAGG-3’  5′-TCCTTGCTCACGCTTACGGA-3’  5′-HEX-GGCTTTGACGCGGGCGTAGT-BHQ1–3’ | 132 |
